# Diagnostic Performance of AI-Based Cloud Software Regarding the Detection of Endodontic Findings on CBCT: A Single-Centre Cross-Sectional Validation Study

**DOI:** 10.3390/jcm15124839

**Published:** 2026-06-22

**Authors:** Maythem Al Fartousi, Arthur Buscot, Christian Ralf Gernhardt

**Affiliations:** 1 Private Practice, Landauer Str. 16, 76185 Karlsruhe, Germany; info@endo-karlsruhe.de; 2 University Outpatient Clinic for Conservative Dentistry and Periodontology, Department of Dentistry, Medical Faculty, Martin-Luther-University Halle-Wittenberg, Magdeburger Strasse 16, 06112 Halle, Germany; 3 Private Practice, Weisenhausdamm 7, 38100 Braunschweig, Germany; info@buscot.de

**Keywords:** artificial intelligence, deep learning, cone-beam computed tomography, endodontic diagnosis, endodontics, apical periodontitis, root canal treatment, diagnostic accuracy, periapical lesion

## Abstract

**Background/Objectives**: The aim of the present investigation was to validate the diagnostic performance of the AI-based dental cloud software Diagnocat^®^ AIS (Version 1.0 (UDI: 860010268018), DGNCT LLC, Miami, FL, USA) regarding the detection possibilities of seven different endodontic findings on cone-beam computed tomography (CBCT) against a multi-rater consensus reference standard, and to characterize its calibration, threshold-optimized performance and clinical utility. **Methods**: 358 root-canal-treated teeth from 167 CBCT scans (167 patients) were retrospectively evaluated at a single private dental practice. From initially included 383 root-canal-treated teeth from 177 patients, 358 (93.5%) were recognized by the AI tool and entered the primary analysis. Two experienced dentists with a clinical focus on endodontics independently graded each tooth and disagreements were adjudicated by a senior expert. Seven different endodontic findings were evaluated: (i) apical (periapical) lesion; (ii) short root-canal filling (apical filling end >2 mm short of the radiographic apex); (iii) voids/lacunae in the root-canal filling; (iv) missed (un-instrumented/un-filled) canal; (v) overfilled root-canal filling (apical extrusion); (vi) apicoectomy (resected root apex with or without retrograde filling); and (vii) coronal restoration with a full-coverage crown. Diagnocat^®^ output was binarized at the manufacturer-fixed 0.50 probability threshold; sensitivity, specificity, predictive values, accuracy, area under the curve AUC (ROC), Cohen κ and Gwet AC1 were computed with 95% cluster-bootstrap confidence intervals (cluster = scan). Threshold optimization, probability calibration, GEE-based subgroup analyses, and decision-curve analysis were pre-specified. **Results**: Diagnostic performance varied by finding. AUCs were 0.984 for missed canal, 0.917 for overfilled root canal, 0.902 for short root filling, 0.893 for crown, 0.864 for apical lesion, 0.857 for apicoectomy and 0.761 for voids in the root filling. Apical-lesion sensitivity rose from 33.6% for sub-millimeter lesions to ≥80% for lesion measuring 1–5 mm. Re-tuning the decision threshold raised missed-canal sensitivity from 69.6% to 97.5%. Decision-curve analysis confirmed positive benefits for missed canal and root-filling-quality findings. **Conclusions**: The AI tool Diagnocat^®^ can be recommended as a focused screening adjunct in CBCT-based endodontic interpretation for missed canals, crowns, and gross root-filling-quality flaws. Sub-millimeter apical lesions and several less common findings (resorption, instrument fragment, retrograde filling) remain outside the reliable performance envelope of the current platform.

## 1. Introduction

Apical periodontitis remains the most prevalent inflammatory disease of the periradicular tissues and the most common adverse outcome of endodontic disease and treatment [1,2,3]. Recent meta-analyses estimate that half of the adult population worldwide showed at least one tooth with apical periodontitis, with the lesion being approximately ten times more frequent in root-canal-treated (RCT) than in untreated teeth (39% versus 3% at the tooth level) [2,4]. The persistence or de-novo emergence of apical periodontitis after endodontic therapy is multifactorial, but the most consistently identified treatment side risk factors are the technical quality of the root canal filling—the used materials, its length, density, and apical adaptation—and the presence of un-instrumented (missed) canals [5,6,7]; the latter alone account for almost half of molar retreatment cases and have been associated with apical periodontitis prevalences exceeding 90% in dedicated CBCT cohorts [8,9]. Recently published studies showed that the risk for apical periodontitis is remarkable high if treatment of the mb2 is missing in upper molars [10].

Cone-beam computed tomography (CBCT) has become the imaging modality of choice when two-dimensional radiography is insufficient to characterize complex endodontic anatomy or post-treatment pathology [11,12]. Compared with intra-oral periapical radiographs, CBCT doubles the detection rate of periapical lesions and substantially improves the diagnosis of root resorption, vertical root fracture, root-canal fillings extruded into adjacent anatomical structures and morphological canal aberrations [12,13]. The Estrela CBCT periapical index (CBCT-PAI) provides a six-grade structural classification of periapical bone destruction with reported inter-observer kappa values of 0.86–0.96 and is now a widely used reference for periapical disease severity [13]. The accompanying clinical benefit of CBCT, however, is paid for in scan volume: a typical full arch volume contains 200–600 axial slices and several dozen RCT teeth across a heterogeneous case mix, generating a workload that is widely acknowledged to challenge consistent and exhaustive interpretation in routine practice [11,12].

Artificial intelligence (AI), particularly convolutional neural networks (CNNs), has emerged as an objective, reproducible, and time-saving aid for radiographic interpretation in endodontics [14,15,16]. Several research-grade CNN models have already been validated for detecting periapical lesions on CBCT, with reported sensitivities of 0.85–0.93 and AUC values approaching 0.98 in selected datasets [17,18,19]. More recently, these methods have been incorporated into commercial cloud-based platforms accessible from standard clinical workstations. One of the most widely used systems in Europe is Diagnocat AIS (DGNCT LLC, Miami, FL, USA), which analyzes CBCT, panoramic, and intra-oral radiographs through a multi-module CNN pipeline that automatically performs tooth recognition, anatomical segmentation, and finding-level prediction, providing the output as a continuous probability score [20].

Several recent studies have begun to characterize the performance of Diagnocat^®^ for endodontic findings, but coverage remains uneven [21,22,23,24]. On panoramic radiographs, accuracy is modest and finding-dependent, with reported sensitivities for short root canal fillings, voids and overextended root canal fillings ranging from 0.32 to 0.91 [25]. On CBCT—for which the platform was specifically optimized—the only published evaluation to date demonstrates an overall accuracy above 94% across endodontic-treatment quality parameters but rests on a relatively small case sample, did not address sample-size–dependent issues such as cluster correlation between teeth from the same scan, and did not quantify the impact of either CBCT image quality or the manufacturer-fixed 0.50 decision threshold on diagnostic performance [25]. Published Diagnocat^®^ CBCT validation studies have not yet reported probability calibration, threshold-optimized metrics, or decision-curve net benefit, even though these are now regarded as essential components of rigorous diagnostic AI evaluation [15,26]. CBCT image quality is a particular concern in this context: AI performance on CBCT is known to be sensitive to noise and metal-artefact load, and the typical signal-to-noise ratio (SNR) in a private-practice case mix varies several-fold between very-good and high-noise volumes [27].

The present study was designed to fill these gaps. Using a single-center, retrospective cross-sectional design and a multi-rater consensus reference standard adjudicated by a senior endodontic expert, we evaluated Diagnocat^®^ AI [20,23] on 358 root-canal-treated teeth across 167 CBCT scans against seven endodontic findings, and additionally provide descriptive data on four findings for which the AI does not currently produce a usable output. The aims of the study were:

1. to quantify the per-tooth diagnostic accuracy of Diagnocat^®^ (sensitivity, specificity, predictive values, accuracy, AUC, Cohen’s κ, and Gwet’s AC1) for apical lesion, short root filling, voids in the root filling, missed canal, overfilled root filling, apicoectomy and crown—using the manufacturer-fixed 0.50 probability cut-off and reporting all confidence intervals as cluster bootstraps that account for within-scan correlation between teeth;
2. to characterize the calibration of the AI probability score and to evaluate the gain in sensitivity and specificity that can be obtained by retuning the decision threshold in a data-driven manner.
3. to test whether diagnostic performance is altered by jaw, tooth class, field of view (FOV) and CBCT image quality (SNR category), with formal interaction testing in a generalized-estimating-equations framework.
4. to assess the dependence of apical-lesion detection on lesion size and Estrela CBCT-PAI severity class; and
5. to translate these diagnostic-accuracy outcomes into a measure of clinical utility through decision-curve analysis.

By combining a clinically representative cohort, methodologically transparent statistics and a comprehensive multi-finding analysis, the study aims to clarify where Diagnocat^®^ is currently suitable to act as a screening adjunct in endodontic CBCT interpretation and where its outputs require cautious clinical interpretation.

## 2. Materials and Methods

### 2.1. Study Design

**Figure 1:**
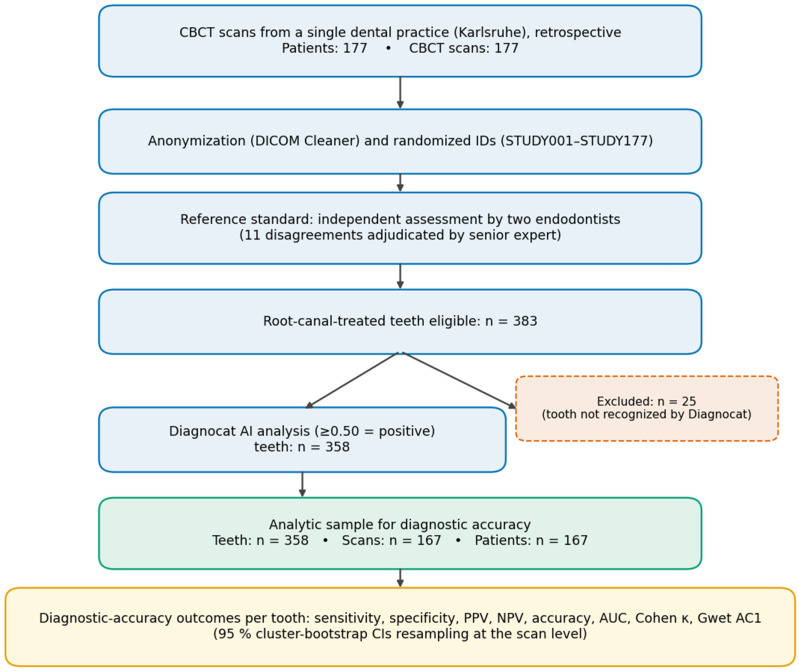
STARD 2015 flow diagram. Patients (n = 177) and CBCT scans (n = 177) from a single dental practice underwent DICOM anonymization, randomization, and re-numbering (STUDY001–STUDY177). Two endodontists evaluated each scan independently; eleven disagreements were adjudicated by a senior expert (gold standard). Of 383 root-canal-treated teeth, Diagnocat^®^ AI processed 358 (93.5%); 25 teeth not recognized by the software were excluded from the primary analyses (sensitivity analyses including these teeth as AI-negative are reported in Supplementary Table S2). The analytic sample for diagnostic-accuracy outcomes therefore comprised 358 teeth across 167 scans and 167 patients. Ten scans were excluded because none of the root canal-treated teeth present in these scans were recognized by the AI software, resulting in the absence of tooth-level AI outputs required for comparison with the reference standard.

We conducted a retrospective single-center cross-sectional diagnostic-accuracy study comparing the AI-based dental software Diagnocat^®^ AIS (DGNCT LLC, Miami, FL, USA) Version 1.0 (UDI: 860010268018). against a multi-rater consensus reference standard for the detection of seven endodontic findings on cone-beam computed-tomography (CBCT) images. Reporting follows the Standards for Reporting of Diagnostic Accuracy Studies 2015 (STARD 2015) [26]; The manufacturer had no role in study design, data acquisition, image interpretation, reference-standard generation, statistical analysis, manuscript preparation, or publication decisions. The corresponding flow diagram is displayed within Figure 1.

### 2.2. Ethics

Ethical approval was issued by the Ethics Committee of the Medical Faculty of the Martin-Luther-University Halle-Wittenberg (reference number 2025-214, 4 November 2025). All CBCT data were fully anonymized before any project-related analysis, the requirement for written informed consent was waived. The study was conducted in accordance with the Declaration of Helsinki (1964, latest revision 2013) and the current local data-protection regulations.

### 2.3. Source Population, Inclusion and Exclusion

CBCT examinations were retrieved from the routine archive of a single private dental practice in Karlsruhe, Germany, covering examinations performed for diverse clinical indications (i.e., implant planning, endodontic re-evaluation, surgical planning). A scan was eligible for inclusion if it contained at least one root-canal-treated (RCT) tooth, defined radiographically by the presence of radiopaque material occupying part or the full length of one or more root canals. There was no restriction on patient age, sex, jaw region, or time since the index endodontic treatment. Scans of insufficient diagnostic quality (severe motion artefact, beam-hardening artefact rendering the apical third unevaluable, or technical truncation) were excluded prior to anonymization.

The unit of analysis was the tooth. All RCT teeth visible within the field of view of an eligible scan were included; unrooted teeth, deciduous teeth, and teeth represented only in the truncated periphery of the volume were excluded. The final eligibility pool comprised 383 RCT teeth from 177 patients across 177 CBCT scans (one anonymized scan ID was initially associated with two patient identifiers, and one further patient identifier carried whitespace inconsistencies; both were reconciled to a single patient chart during data cleaning).

### 2.4. CBCT Acquisition

All CBCT scans were acquired using a Veraviewepocs 3D R100 system (J. Morita Mfg. Corp., Kyoto, Japan). Scans obtained with 4 × 4 cm and 4 × 8 cm fields of view were reconstructed with a voxel size of 125 µm, whereas scans obtained with an 8 × 10 cm field of view were reconstructed with a voxel size of 160 µm.

All CBCT examinations were obtained on the same CBCT used in routine clinical practice at the contributing dental practice (single device, single operator team), with a tube potential of 90 kV and clinical exposure protocols selected by the operator based on diagnostic question and field-of-view (FOV). Three FOVs were represented in the dataset: 4 × 4 cm (high-resolution single-quadrant scans), 10 × 4 cm (extended arch scans) and 10 × 8 cm (full-jaw scans), with voxel sizes specific to the manufacturer’s protocol for each FOV. Reconstructed volumes were exported as DICOM files for downstream processing.

### 2.5. Anonymisation and Randomisation

For every CBCT examination, a folder of DICOM slices was generated. All DICOM headers were anonymized using DicomCleaner™ (PixelMed Publishing, Bangor, PA, USA), removing all 18 categories of HIPAA Protected Health Information (PHI) and any institution-, operator- or device-level identifiers. The anonymized series were then re-numbered and additionally randomized in their file-system order using Bulk Rename Utility Version 4.1.0.1 (TGRMN Software, Fullarton, Australia). Final dataset identifiers were assigned as STUDY001–STUDY177 to break any chronological ordering of the original archive. Anonymization occurred prior to both the human reference-standard assessment and the index AI evaluation, so that every assessor (human and machine) was blinded to clinical context, patient identity, and acquisition order.

### 2.6. Reference Standard (Gold Standard)

Two dentists with a clinical focus on endodontics independently evaluated each anonymized CBCT volume using the same multiplanar viewer i-Dixel (J. Morita Mfg. Corp., Kyoto, Japan). Rater 1 had six years and Rater 2 ten years of dedicated clinical experience in endodontics. Both readers routinely use CBCT imaging in daily clinical practice whenever indicated. Preliminary ruling interrater agreement between readers 1 and 2 was excellent. Percentage agreement ranged from 98.8% to 100% across all evaluated findings. Cohen’s κ values ranged from 0.83 to 1.00 for the clinically relevant findings. Only eleven disagreements were observed across the entire dataset prior to adjudication, comprising eight resorption assessments, one root-filling-length assessment, and two root-filling-density assessments.

Each rater reviewed all volumes in randomized order without access to the other rater’s assessment, the AI output, or any clinical metadata. For every RCT tooth, raters recorded the presence (1) or absence (0) of each of eleven endodontic findings (Section 2.8) plus the number of anatomical canals and the Estrela CBCT periapical index (s0–s6) [13]. For positive apical lesions, the maximum lesion diameter (mm) was measured in the plane of greatest extent. In 1 of 358 teeth in the analytic sample (0.3%), the Estrela class (s4) and the binary apical-lesion label diverged after consensus adjudication. The binary label was used for the primary diagnostic-accuracy analyses (Section 3.2), while the Estrela class was retained for the severity-stratified sub-analyses (Section 3.5); this single discrepancy is the source of the off-by-one count between Estrela s4 (n = 45) and the apical-lesion-positive count for Estrela s4 (n = 44) (Section 3.1 and Section 3.5).

Disagreements between Readers 1 and 2 were resolved by a senior endodontic expert, whose adjudication served as the binding reference standard. The adjudicator holds a university professorship in Endodontics and is a former President of the German Society of Endodontology and Dental Traumatology (DGET). whose adjudication served as the binding reference. In the present cohort, eleven disagreements occurred in total: eight concerned suspected root resorptions, one concerned root-canal filling length (too short/too long), and two concerned insufficient root-canal filling density. The consensus assessment of all 383 teeth—in which the rater-1/rater-2 agreement was retained where concordant and the senior-expert adjudication was substituted otherwise—constituted the gold.

### 2.7. Index Test (Diagnocat^®^ AI)

After all human assessments were finalized, the anonymized CBCT volumes were uploaded in batch to the Diagnocat^®^ cloud platform via the manufacturer’s web interface. The platform automatically performed tooth recognition, anatomical segmentation, and finding-level prediction. For every RCT tooth that the platform recognized, a continuous probability score in the range 0.00–0.99 was returned for each modelled finding. In line with the manufacturer’s documentation and our pre-specified protocol, AI predictions with probability ≥0.50 were treated as positive and <0.50 as negative for the primary binary analyses [20]; threshold-sensitivity analyses across alternative cut-offs (0.30, 0.70, Youden-optimal) are reported separately (for additional information see Section 2.10).

Twenty-five of the 383 eligible RCT teeth were not recognized as teeth by Diagnocat^®^. These teeth were excluded from the primary diagnostic-accuracy analyses (analytic sample n = 358) but were retained for a worst-case sensitivity analysis in which they were treated as AI-negative for every finding (Section 2.10, Supplementary Table S2).

The Diagnocat^®^ operator who uploaded the volumes did not interact with the platform’s output for the human reference standard, and the platform was not given access to the human assessments at any stage; the index test was therefore obtained in full blinding to the gold standard.

### 2.8. Endodontic Findings Analysed

The reference standard catalogued twelve endodontic findings. Diagnocat^®^ returned non-trivial probability output for seven of these, which constituted the analyzable set: (i) apical (periapical) lesion; (ii) short root-canal filling (apical filling end > 2 mm short of the radiographic apex); (iii) voids/lacunae in the root-canal filling; (iv) missed (un-instrumented/un-filled) canal; (v) overfilled root-canal filling (apical extrusion); (vi) apicoectomy (resected root apex with or without retrograde filling); and (vii) coronal restoration with a full-coverage crown.

Five further findings—lateral periapical lesion, internal resorption, external resorption, retrograde root-canal filling, and instrument fragment—could not be evaluated for diagnostic accuracy: lateral periapical lesion produced a uniformly zero probability across all 358 analyzed teeth, and the remaining four findings had no corresponding AI output category. These are reported descriptively only (Section 3.8).

In addition, Diagnocat^®^ returned an AI estimate of the number of anatomical canals per tooth, which was compared against the consensus canal count (Section 3.9, Figure S2).

### 2.9. CBCT Image-Quality Quantification

Image quality was quantified independently of the human reference standard, on the anonymized volumes, using ImageJ (Version 1.54s, National Institutes of Health, Bethesda, MD, USA). For each volume, a circular region of interest (ROI) of standardized size was placed in a homogeneous soft-tissue/cancellous-bone area free of metallic artefacts. Three repeated ROI placements were performed per scan; for each placement, the mean grey-value (MEAN) and the standard deviation of grey-values (SD) were extracted. The averaged SD across the three measurements served as the noise estimator, and the signal-to-noise ratio (SNR) of the volume was defined as MEAN/SD. Volumes were stratified into four manufacturer-suggested image-quality categories using fixed cut-offs: SNR > 10 (very good), 6 ≤ SNR ≤ 10 (clinically good), 3 ≤ SNR < 6 (moderate noise), and SNR < 3 (high noise).

### 2.10. Statistical Analysis

All statistical analyses were performed in Python 3.12 (Python Software Foundation, Wilmington, DE, USA) using NumPy 2.4, pandas 2.3, scikit-learn 1.8, statsmodels 0.14 and SciPy 1.17 (All: Python Software Foundation, Wilmington, DE, USA); figures were generated in matplotlib 3.10 (Python Software Foundation, Wilmington, DE, USA).

#### 2.10.1. Primary Diagnostic-Accuracy Outcomes

For each of the seven analyzable findings, a 2 × 2 contingency table was constructed at the per-tooth level using the manufacturer-fixed 0.50 cut-off, and the following metrics were derived: sensitivity, specificity, positive predictive value (PPV), negative predictive value (NPV), accuracy, Youden’s J, and Cohen’s κ. Because Cohen’s κ is unstable for highly skewed prevalence, Gwet’s first-order agreement coefficient AC1 was reported alongside κ [28]. The continuous Diagnocat^®^ probability score was used to compute the area under the receiver-operating-characteristic curve (AUC).

Because each scan could contribute multiple teeth (median one tooth per scan, range 1–8), per-tooth observations were not statistically independent. To accommodate this, all 95% confidence intervals (CIs) for the primary metrics were obtained by cluster bootstraps with two thousand replications, in which scans (STUDY_ID) were resampled with replacement and all teeth belonging to a sampled scan were carried into the bootstrap replicate. CIs are presented as the 2.5th–97.5th percentiles of the bootstrap distribution. AUC CIs were obtained by the same cluster-bootstrap procedure with one thousand replications.

#### 2.10.2. Threshold and Calibration Analysis

To examine the impact of the manufacturer-fixed cut-off, diagnostic metrics were re-computed at three additional thresholds—0.30, 0.70, and the Youden-optimal threshold derived from the empirical ROC (the cut-off maximizing sensitivity + specificity − 1)—for each finding. Probability calibration was assessed using the Brier score and a logistic-recalibration model (calibration intercept and slope obtained by regressing the gold-standard label on logit (Diagnocat^®^ probability)) [29]; reliability diagrams used quantile-based binning (10 bins where positives and negatives both exceeded 60 cases, 5 bins otherwise).

#### 2.10.3. Subgroup Analyses

Pre-specified subgroup analyses were performed for each finding × subgroup combination across (i) jaw (maxilla vs. mandible), (ii) tooth class (anterior, premolar, molar—collapsed from FDI groups), (iii) FOV (4 × 4 vs. 10 × 8 cm; the 10 × 4 stratum, n = 2 teeth, was excluded), and (iv) image-quality category (very good/clinically good/moderate/high noise). Sensitivity and specificity within each subgroup level were estimated with the same cluster-bootstrap CI procedure (one thousand replications). Statistical heterogeneity of the AI probability across subgroup levels was assessed by generalized estimating equations (GEE) logistic regression with the gold-standard label as outcome, the AI probability score as the principal predictor, the subgroup as a factor, and an AI × subgroup interaction term; an exchangeable working correlation structure was specified, with scan as the cluster identifier and Wald-test–based robust (sandwich) standard errors. The reported *p*-value tests the joint contribution of all interaction terms. However, because several subgroup strata contained sparse event counts and, in some analyses, complete or quasi complete separation, interaction tests were considered exploratory. Non convergent or non-estimable interaction models were not interpreted.

#### 2.10.4. Apical-Lesion Deep-Dive Analyses

Apical-lesion sensitivity was further stratified by Estrela CBCT-PAI class (s0–s6 individually and grouped as s0–s3 vs. s4–s6) and by lesion-diameter strata (<1 mm, 1–<3 mm, 3–<5 mm, ≥5 mm), with cluster-bootstrap CIs. The continuous association between lesion diameter and AI detection (gold-positive teeth only) was modelled by GEE logistic regression with detection as outcome, diameter as continuous predictor, exchangeable working correlation, and scan as cluster; the corresponding odds ratio per +1 mm of lesion diameter is reported with its 95% CI and Wald *p*-value.

#### 2.10.5. Decision-Curve Analysis

To translate the diagnostic-accuracy results into a quantitative measure of clinical utility, decision-curve analysis (DCA) was performed for the four findings of greatest clinical relevance (apical lesion, missed canal, short root filling, overfilled root canal) [30]. Net benefit was computed as NB(p_t) = TP/n − FP/n × p_t/(1 − p_t) and plotted across the clinically plausible threshold range 0.05–0.50, alongside the “treat all” and “treat none” reference strategies.

#### 2.10.6. Sensitivity Analyses

Two pre-specified sensitivity analyses were performed (Supplementary Table S2): (a) a worst-case full-cohort analysis (n = 383) in which the 25 teeth not recognized by Diagnocat^®^ were treated as AI-negative for every finding; and (b) a per-patient analysis in which one randomly selected tooth per scan (n = 167 teeth) was retained, eliminating within-scan clustering by design.

#### 2.10.7. Reporting Conventions and Missing Data

All *p*-values are two-sided and unadjusted; given the descriptive nature of the diagnostic-accuracy outcomes, no formal multiplicity adjustment was applied for the seven primary findings. Where subgroup-level estimates were derived from < 10 teeth, the corresponding cell is marked “—” rather than reporting unstable estimates. There were no missing reference-standard labels for the seven analyzable findings; 22 of 383 teeth (5.7%) had a missing AI canal-count estimate (used only for the descriptive analysis in Figure S2); image-quality categorization was complete for all teeth. A two-sided α of 0.05 was used as the nominal threshold for inferential subgroup tests, recognizing that all such tests were exploratory.

## 3. Results

### 3.1. Cohort and Tooth-Level Characteristics

A total of 177 cone-beam computed-tomography (CBCT) datasets from 177 patients (yielding 383 root-canal-treated teeth) were included in the eligibility pool. Diagnocat^®^ recognized 358 of 383 teeth (93.5%); 25 teeth (6.5%) could not be processed by the AI software and were excluded from the diagnostic-accuracy analyses, in line with the pre-specified protocol. The analytic sample therefore comprised 358 teeth from 167 scans (167 patients), with a median of one tooth per scan (interquartile range 1–3, range 1–8). Maxillary teeth predominated (n = 234, 65.4%), and molars represented the largest tooth class (n = 168, 46.9%). Most scans were acquired with a 10 × 8 cm field of view (FOV; 80.7%); image-quality stratification using the signal-to-noise ratio (SNR; mean grey value/SD) classified 28.8% of teeth as “very good”, 48.9% as “clinically good”, 19.3% as “moderate noise”, and 3.1% as “high noise”. Apical lesions according to Estrela CBCT-PAI were present in 72.1% of teeth, with a near-uniform distribution across active-disease classes s1–s4 (Figure 1, Table 1). Recruitment, anonymization, reference standard, and analytic-sample derivation are summarized in Figure 1.

### 3.2. Overall Diagnostic Performance

**Figure 2:**
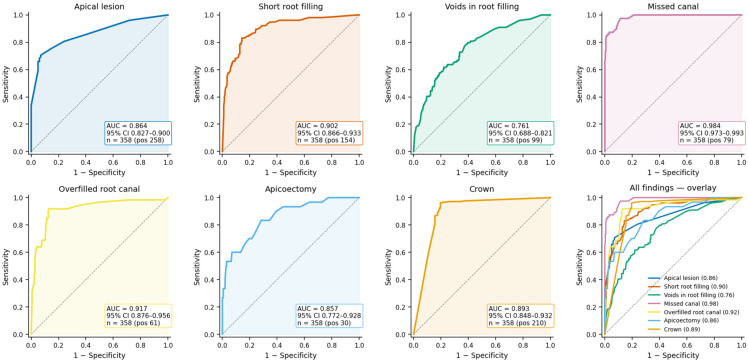
Receiver-operating-characteristic curves for the seven analyzable findings. Each panel shows the ROC curve derived from the continuous Diagnocat^®^ probability score with the corresponding area under the curve (AUC) and 95% cluster-bootstrap confidence interval (one thousand replications). The bottom-right panel overlays all seven findings for direct comparison. AUCs ranged from 0.761 (voids in root filling) to 0.984 (missed canal). The dotted diagonal line in each ROC panel represents the line of no discrimination.

**Figure 3:**
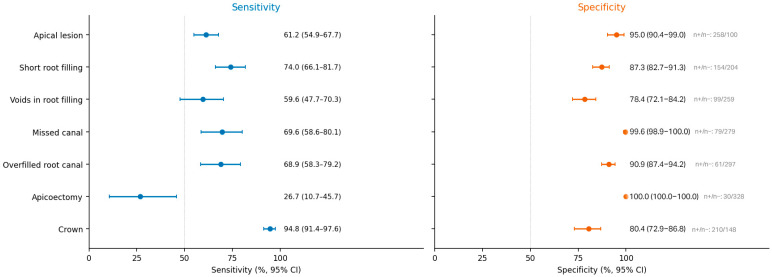
Forest plot of sensitivity (**left**) and specificity (**right**) for all seven findings, with 95% cluster-bootstrap CIs. The dotted vertical line marks the 50% reference. Annotation on the right-hand panel shows the absolute number of gold-positive (n+) and gold-negative (n−) teeth contributing to each finding.

**Figure 4:**
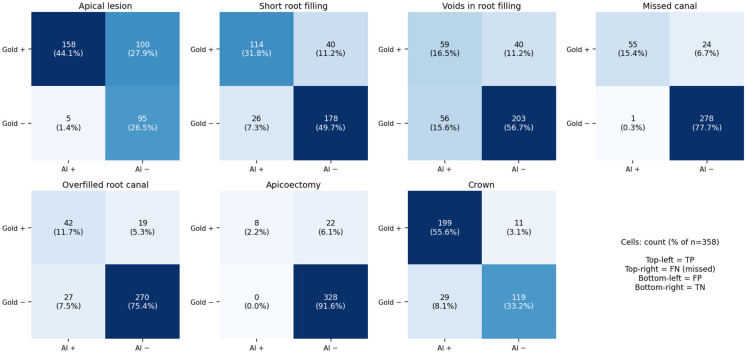
Confusion-matrix heat-maps for the seven analyzable findings. Each cell is annotated with the absolute count of teeth and the corresponding proportion of the analytic sample (n = 358). Darker blue color intensity reflects a larger proportion of the sample falling into that cell.

For the seven analyzable findings, per-tooth diagnostic accuracy of Diagnocat^®^ against the consensus reference standard is summarized in Table 2 and Figure 2, Figure 3 and Figure 4. Cluster-bootstrap 95% confidence intervals (CIs) were obtained by resampling at the scan level (two thousand replications), reflecting the within-scan correlation of teeth.

Two findings reached high overall accuracy: missed canal detection (AUC 0.984, 95% CI 0.972–0.993; sensitivity 69.6% [58.7–79.4], specificity 99.6% [98.8–100.0], PPV 98.2% [93.8–100.0], NPV 92.1% [88.8–94.9]; Cohen κ 0.773, Gwet AC1 0.899) and crown detection (AUC 0.893, 95% CI 0.851–0.932; sensitivity 94.8% [91.3–97.7], specificity 80.4% [73.5–86.8]; κ 0.765). Short root filling (AUC 0.902; sensitivity 74.0% [65.8–81.4], specificity 87.3% [83.0–91.5]; κ 0.620) and overfilled root canal (AUC 0.917; sensitivity 68.9%, specificity 90.9%; κ 0.568) showed strong AUCs together with moderate-to-substantial agreement. Apical-lesion detection combined a high AUC (0.864) and very high specificity (95.0% [90.0–99.0]) with a comparatively low sensitivity (61.2% [54.9–67.5]) at the manufacturer-fixed 0.50 threshold, yielding only fair agreement (κ 0.436). Voids in the root filling (AUC 0.761; κ 0.362) and apicoectomy (AUC 0.857; sensitivity 26.7% [9.5–45.2], specificity 100.0%) were the weakest of the analyzable findings.

### 3.3. Threshold Analysis and Probability Calibration

Because the manufacturer-fixed 0.50 cut-off is not necessarily optimal in clinical practice, we evaluated diagnostic performance at three additional thresholds (0.30, 0.70 and the Youden-optimal value derived from the ROC) (Table 3). Lowering the threshold yielded clinically relevant gains in sensitivity for several findings without a large penalty in specificity. The most striking shift was observed for missed canals: at the Youden-optimal threshold of 0.09, sensitivity rose from 69.6% to 97.5% while specificity remained at 88.5%, raising Youden J from 0.69 to 0.86. Similar gains were seen for overfilled root canal (sensitivity 68.9 → 91.8%; threshold 0.15) and apicoectomy (26.7 → 83.3%; threshold 0.16), at the cost of a marked drop in specificity for the latter (100 → 71.6%). Because these thresholds were derived and evaluated within the same dataset, the resulting performance estimates should be interpreted as exploratory and may be optimistically biased. For apical lesion, the optimum was below the lowest non-zero output of the network (threshold 0.07), suggesting that the manufacturer threshold is set too conservatively for this finding. Crown detection was already near optimum at 0.50.

**Figure 5:**
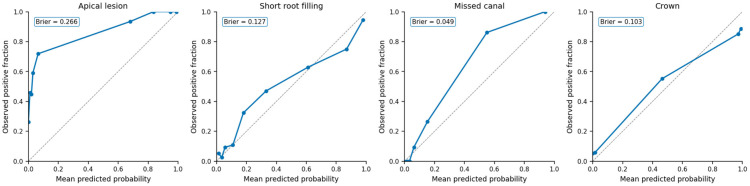
Probability-calibration (reliability) diagrams for the four most clinically relevant findings (apical lesion, short root filling, missed canal, crown). The diagonal dashed line indicates perfect calibration; deviations above the diagonal indicate that the AI under-predicts the true positive fraction. Brier scores (lower is better) are shown in the top-left of each panel.

Probability calibration is presented in Figure 5 for the four most clinically relevant findings, with full Brier scores and logistic recalibration parameters in Supplementary Table S1. Probability-score distributions for all analyzable findings are shown in Supplementary Figure S1. Missed canal demonstrated excellent discrimination but only moderate calibration. (Brier 0.049) and crown (Brier 0.103). Apical-lesion probabilities were systematically under-predicted across the entire range (Brier 0.266; observed positive fraction 0.45 at predicted probability 0.005, and 1.00 at predicted probabilities ≥ 0.65), which explains the simultaneous combination of high specificity and low sensitivity at the default threshold.

### 3.4. Subgroup Analyses by Anatomical Site, FOV, and Image Quality

Subgroup performance was evaluated across (i) jaw, (ii) tooth class (anterior, premolar, molar), (iii) FOV (4 × 4 vs. 10 × 8 cm; the 10 × 4 stratum was excluded with n = 2) and (iv) SNR-based image-quality category. GEE logistic regression (exchangeable working correlation; cluster = scan) was used to evaluate for an interaction between subgroup and the AI probability score (Table 4). Specificity was uniformly high across all subgroups for the strong-performing findings (missed canal, crown). Heterogeneity in sensitivity was, however, observed.

**Figure 6:**
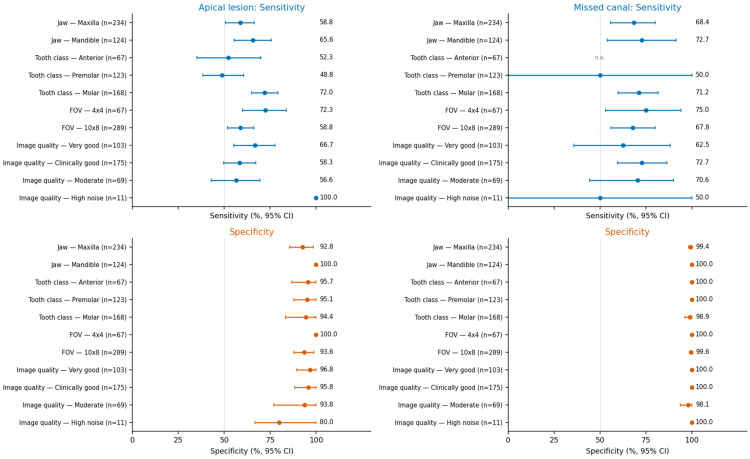
Subgroup forest plot of sensitivity (**top row**) and specificity (**bottom row**) for the two headline findings—apical lesion (**left**) and missed canal (**right**). Each row corresponds to one subgroup level (jaw, tooth class, FOV, and image-quality category) annotated with the stratum size; horizontal bars show the 95% cluster-bootstrap CI. The dotted vertical line marks the 50% reference.

For apical-lesion detection, interaction analysis differed significantly by jaw (interaction *p* = 0.038), with maxillary teeth showing 58.8% (50.8–66.4) and mandibular teeth 65.6% (54.5–75.5). Sensitivity was substantially higher in molars (72.0%) than in premolars (48.8%) and anterior teeth (52.3%), although the formal interaction was not significant (*p* = 0.78). Sensitivity was also higher with the 4 × 4 cm FOV (72.3%) than the 10 × 8 FOV (58.8%). For voids in root filling, sensitivity was higher in the mandible (67.4 vs. 52.8% in the maxilla; *p* = 0.047). For short root filling and overfilled root canal, FOV-stratified specificity was significantly higher with the 10 × 8 acquisition. Sensitivity for missed canal could not be reliably estimated for anterior teeth because no anterior tooth was gold-positive; among premolars, prevalence was very low (n = 6 positive), producing a wide CI (50.0% [0–100]). Performance for the two headline findings (apical lesion and missed canal) is visualized across all subgroups in Figure 6.

### 3.5. Apical Lesions: Estrela Class and Lesion Size

Among the 258 apical-lesion-positive teeth, AI sensitivity increased with disease severity (Table 5, Figure S3): from 24.4% in Estrela s1 (small structural change) to 61.2% (s2), 82.6% (s3), 86.4% (s4) and furthermore decreased to 78.6% (s5) and 75.0% (s6). Pooled into the Estrela groupings, sensitivity for early disease (s1–s3) was 54.1% (95% CI 46.8–61.4) and for established periapical osteitis (s4–s6) 83.9% (75.0–92.4).

**Figure 7:**
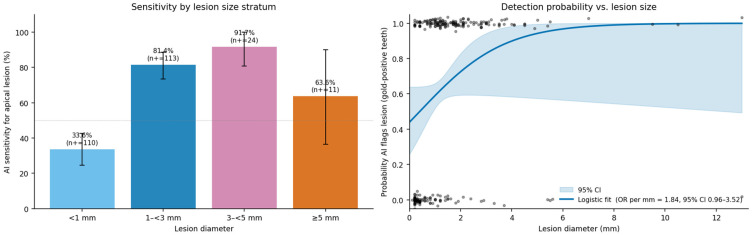
Apical-lesion detection by lesion size. Left panel: AI sensitivity (with 95% cluster-bootstrap CI) within four lesion-diameter strata (sub-millimeter lesions are mostly missed; 1–5 mm lesions show high sensitivity; the ≥5 mm stratum is small). Right panel: GEE logistic regression fit (exchangeable working correlation; cluster = scan) of detection probability on lesion diameter, with shaded 95% pointwise CI. The reported odds ratio per +1 mm corresponds to the detection-probability gradient on the linear-predictor scale.

Lesion-size strata (Table 6, Figure 7) showed a similar dependence: AI sensitivity was 33.6% (24.5–42.5) for sub-millimeter lesions, 81.4% (73.4–88.6) for 1–<3 mm lesions, and 91.7% (80.7–100.0) for 3–<5 mm lesions. The sensitivity in the ≥5 mm stratum dropped to 63.6% (36.4–90.0) but rests on only 11 teeth; visual inspection of the discordant cases suggested that these very large radiolucencies extended beyond the labelled lesion centroid in several scans. In a GEE logistic regression of detection vs. lesion diameter (gold-positive teeth, cluster = scan), the odds of AI detection per +1 mm of lesion diameter was 1.84 (95% CI 0.96–3.52, *p* = 0.067), suggesting a positive size–detection gradient that did not, however, formally reach significance because of high inter-tooth variability.

### 3.6. Influence of CBCT Image Quality

Image quality, expressed via the SNR category, did not produce a uniform decrement in performance. For findings, whose detection depends on subtle low-contrast features—voids in the root filling and apical lesions—sensitivity declined modestly with worsening SNR, but the GEE interaction term was not significant for apical lesion (*p* = 0.997) or for voids (*p* = 0.618). For findings dominated by high-contrast structures (missed canals, crown, short root filling, overfilled root canal), specificity remained ≥ 95% across all SNR categories; the highest-noise stratum was sparse (n = 11 teeth) and contributed little. Because several SNR strata contained sparse event counts and, for some findings, complete or quasi-complete separation, the corresponding GEE interaction tests were considered exploratory or non-estimable. Therefore, image-quality subgroup results are reported descriptively and should not be interpreted as formal evidence of SNR-dependent calibration heterogeneity (Table 4). Overall, these data argue against a strong, image-quality-dependent decline in Diagnocat^®^ performance within the typical clinical range encountered.

### 3.7. Results of the Decision-Curve Analysis

**Figure 8:**
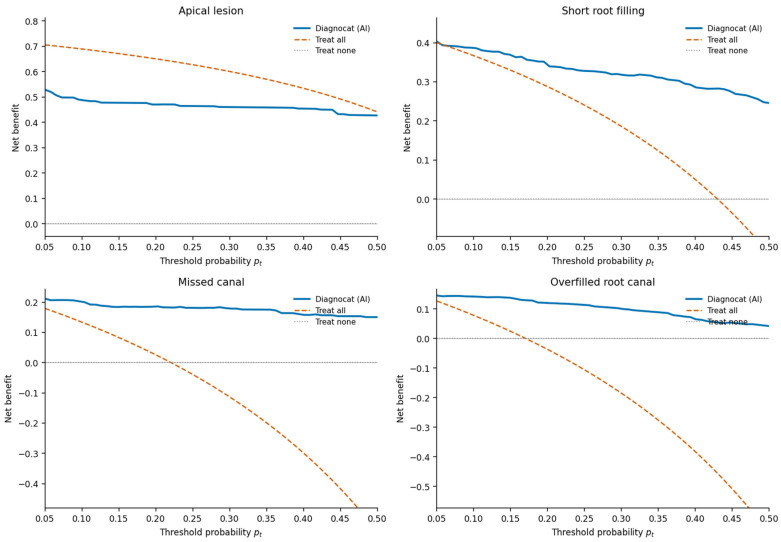
Decision-curve analysis (DCA). Net benefit (TP/n − FP/n × p_t/(1 − p_t)) is plotted over the clinically plausible threshold range 0.05–0.50 for the four findings of greatest interest. Solid blue line = Diagnocat^®^ probability score; orange dashed line = “treat all” reference; grey dotted line = “treat none.” The AI strategy is preferable at thresholds where its line lies above both references.

Net-benefit curves for the four findings of greatest clinical interest are shown in Figure 8. For missed canal, short root filling and overfilled root canal, Diagnocat^®^ provided positive net benefit above both reference strategies (“treat all” and “treat none”) across the entire clinically plausible threshold range (0.05–0.50); the gap was largest above threshold 0.15, where “treat all” loses net benefit because of the false-positive cost. For apical lesion—a finding with high baseline prevalence (72%)—the “treat all” strategy outperformed Diagnocat^®^ across most of the threshold range, consistent with the lower sensitivity of the AI score for early-disease (Estrela s1–s2) lesions; AI offered net benefit only at higher decision thresholds where false-positive costs dominate.

### 3.8. Results of the Findings Without Usable AI Output

For four endodontic findings the Diagnocat^®^ probability output was either uniformly zero (lateral periapical lesion: probability = 0.00 in 358/358 teeth) or not reported by the software (internal resorption [n = 5 gold-positive], external resorption [n = 14], retrograde root filling [n = 16] and instrument fragment [n = 1]). These findings were therefore not amenable to AUC, sensitivity or specificity estimation and are reported here descriptively only. Their absence from the AI output represents the principal scope limitation of the current Diagnocat^®^ module for endodontic CBCT analysis.

### 3.9. Results of the Sensitivity Analyses

Two pre-specified sensitivity analyses confirmed the primary results (Supplementary Table S2). Conservative AI-negative imputation, in which the 25 unrecognized teeth were treated as AI-negative for all findings, produced minimal attenuation of the headline metrics (e.g., missed canal: AUC 0.919 vs. 0.984; sensitivity 64.7% vs. 69.6%; crown: AUC 0.848 vs. 0.893). A per-patient analysis restricted to one randomly selected tooth per scan (n = 167 teeth) reproduced the full-sample point estimates within the bootstrap CIs (e.g., missed canal: AUC 0.983, sensitivity 76.3%; crown: AUC 0.874, sensitivity 93.3%), indicating that the cluster structure had not biased the primary estimates. Anatomical canal-count agreement between Diagnocat^®^ and the consensus standard was high (Spearman ρ = 0.946; weighted κ_linear = 0.885; exact agreement 86.0%; agreement within ±1 canal 99.2%; Figure S2).

The 25 unrecognized teeth were not randomly distributed but were enriched for clinically relevant findings. Consequently, exclusion of these cases may have resulted in modestly optimistic performance estimates in the primary analysis. The conservative AI-negative imputation analysis was performed to assess the potential magnitude of this effect.

## 4. Discussion

### 4.1. Principal Findings

In this single-center cross-sectional diagnostic-accuracy study comprising 358 root-canal-treated teeth from 167 CBCT scans, the AI platform Diagnocat^®^ showed a wide finding-dependent performance spectrum against a multi-rater consensus reference standard. Two findings reached very high overall accuracy: missed canal (AUC 0.984, sensitivity 69.6%, specificity 99.6%, Cohen’s κ 0.77) and crown detection (AUC 0.893, sensitivity 94.8%, κ 0.77). Short root filling (AUC 0.902) and overfilled root canal (AUC 0.917) showed strong discriminative ability, while apical-lesion detection combined a high AUC (0.864) and excellent specificity (95.0%) with a sensitivity of only 61.2% at the manufacturer-fixed 0.50 threshold. Voids in the root filling (AUC 0.761) and apicoectomy (AUC 0.857; sensitivity 26.7%) were the weakest of the seven analyzable findings, and four further endodontic findings—lateral periapical lesion, internal and external resorption, retrograde root filling and instrument fragment—could not be evaluated because Diagnocat^®^ did not return a usable output for them. To the best of our knowledge, this study is the first methodologically rigorous CBCT validation of Diagnocat^®^ that combines cluster-bootstrap inference, threshold and calibration analysis, decision-curve analysis and pre-specified subgroup analyses by jaw, tooth class, FOV and image quality, on a clinically representative case mix.

### 4.2. Comparison with Previous Diagnocat^®^ and Other AI Evaluations on CBCT

Our results align with, and extend, the small but growing body of Diagnocat^®^-on-CBCT evidence. Kazimierczak and colleagues, in the only previously published Diagnocat^®^-on-CBCT validation, reported overall per-tooth accuracies above 94% across endodontic-treatment quality parameters [31]; our headline accuracy for missed canal (93.0%), apicoectomy (93.9%) and overfilled root canal (87.2%) is fully consistent with their reported findings, but our sample is approximately three-fold larger and our analytic framework explicitly accounts for the within-scan correlation of teeth that previous papers have not addressed. The headline AUC for missed canal (0.984) is also among the highest reported for any commercial dental AI on any imaging modality, and lies within the same range as the dedicated 3D-CNN PAL-Net algorithm for periapical lesions (AUC 0.98) [17] and the Hadzic et al. validation for periapical lesion detection (sensitivity 86.7%, specificity 84.3%) [18]. By contrast, Diagnocat^®^’s performance on panoramic radiographs has consistently been weaker, with sensitivities for short root canal fillings, voids and overextended root canal fillings ranging from 0.32 to 0.91—a difference that certainly reflects the volumetric advantage of CBCT for endodontic interpretation [20,25].

The clinically most consequential observation is the marked size-dependence of apical-lesion detection. Sensitivity rose from 33.6% for sub-millimeter lesions to 81.4% for 1–<3 mm lesions and 91.7% for 3–< 5 mm lesions; the same gradient was reproduced for the Estrela CBCT-PAI classes, with sensitivity rising from 24% at s1 to 86% at s4. This pattern mirrors the validation by Hadzic and colleagues, who reported that half of the smallest lesions (PAI score 1, 0.5–1 mm) were missed by their CNN whereas sensitivity for lesions > 1 mm reached 90.4% [18]. CBCT itself is known to lose sensitivity for very small periapical lesions even when interpreted by experienced endodontists—a function of partial-volume averaging at the trabecular-bone scale [12,14]. The size threshold around 1 mm therefore appears to be a property of the CBCT image rather than of the AI alone and represents the most important practical limitation in screening for incipient periapical disease.

### 4.3. Threshold Tuning, Calibration and Clinical Utility

A second clinically actionable observation is that the manufacturer-fixed 0.50 probability cut-off is suboptimal for several findings. For missed canal, lowering the threshold to a Youden-optimal value of 0.09 raised sensitivity from 69.6% to 97.5% while specificity dropped only modestly from 99.6% to 88.5%, increasing Youden J from 0.69 to 0.86. Substantial gains were also observed for overfilled root canal (sensitivity 68.9 → 91.8%; threshold 0.15) and for apicoectomy (26.7 → 83.3%; threshold 0.16), although the latter came at the cost of specificity (100 → 71.6%). The reliability diagrams confirm that Diagnocat^®^ probability scores are systematically under-confident for apical lesions (Brier 0.266, calibration intercept +1.78 on the logit scale)—an observation that explains the simultaneously high specificity and low sensitivity at the default threshold and that argues for either a finding-specific re-calibration or a dedicated screening threshold in clinical use. Methodologically, the combined use of Brier score, calibration intercept/slope, and reliability binning has been recommended for any prognostic or diagnostic prediction model evaluated for clinical deployment [29].

Decision-curve analysis (DCA) translates these properties into clinical utility [30]. For missed canal, short root filling and overfilled root canal, Diagnocat^®^ provided positive net benefit above both the “treat all” and “treat none” reference strategies across the entire 0.05–0.50 threshold range, with the gap widest above threshold 0.15—exactly the regime in which a screening clinician would consider acting on a flagged finding. For apical lesion, the high baseline prevalence (72%) made “treat all” the dominant reference strategy at low thresholds, and Diagnocat^®^ only produced positive net benefit at higher thresholds where false-positive cost dominates. Read together with the Estrela and lesion-size analyses, this suggests that the most promising clinical role for Diagnocat^®^ in periapical-lesion screening is not as an early-disease detector but rather as a confirmatory tool for already established lesions, and that the most concrete near-term gain is in flagging missed canals and gross root-canal-filling-quality issues.

The decision-curve analyses (DCA) should be interpreted within the context of the retrospective cross-sectional design of the present study. Because only anonymized CBCT datasets were available, no information regarding symptoms, treatment decisions, follow-up findings, or clinical outcomes could be incorporated. Consequently, a positive AI finding was interpreted as a trigger for additional clinician review rather than as a direct indication for treatment. The reported net-benefit estimates therefore reflect potential diagnostic utility and not the effectiveness of specific clinical management strategies.

An important limitation of the threshold analyses is that the Youden-optimal cut-offs were derived and evaluated within the same study population. Consequently, the observed improvements in sensitivity and specificity may partly reflect overfitting to the characteristics of the present dataset and may not fully generalize to external populations. Therefore, these thresholds should be regarded as candidate operating points for future investigation rather than clinically validated decision thresholds. Independent external validation and prospective testing are required before implementation in routine clinical practice can be recommended.

### 4.4. Subgroup Heterogeneity, Image Quality and Anatomical Site

Subgroup analyses revealed several patterns relevant to deployment. Apical-lesion sensitivity was higher in the mandible (65.6%) than the maxilla (58.8%), and substantially higher in molars (72.0%) than in premolars (48.8%) or anterior teeth (52.3%); the GEE interaction term reached statistical significance for jaw (*p* = 0.038) but not for tooth class, indicating that the molar advantage may largely be explained by the higher prevalence and, by extension, the higher fraction of larger Estrela-s3/s4 lesions in molars. The mandibular advantage parallels the observation by Hadzic and colleagues that the lower jaw yields higher CBCT specificity than the maxilla, attributed to better radiological accessibility and lower artefact density in the mandible [18]. The 4 × 4 cm FOV produced numerically higher apical-lesion sensitivity (72.3% vs. 58.8% for the 10 × 8 cm FOV); whether this is driven by the higher in-plane resolution of small-FOV protocols or by selection of cases for the small-FOV protocol cannot be disentangled in our retrospective design.

In the present investigation Diagnocat^®^ performance was, reassuringly, not strongly degraded by reductions in image quality within the typical clinical SNR range. Former investigations showed that high-resolution radiographs enhance diagnostic accuracy for AI and human evaluators [32]. Sensitivity for the four high-contrast findings (missed canal, crown, short and overfilled root filling) remained stable across SNR categories, and specificity remained ≥95% even in the moderate-noise stratum. A small subset of high-noise volumes (n = 11) produced unstable estimates with very wide CIs but did not change the overall pattern. The robustness of the AI to image-quality fluctuation is consistent with parallel work showing that CBCT image-quality decrements that are visually obvious to clinicians do not necessarily translate into proportional decrements in CNN performance, particularly for high-contrast targets [28]. It is, however, important to note that a single CBCT device was used in this study and that more aggressive variation in acquisition protocols, different vendors, different reconstruction kernels, and metal artefact load—could affect performance in ways our subgroup analysis cannot rule out.

### 4.5. Discussion of Findings Without Usable AI Output

Diagnocat^®^ returned a uniformly zero probability for lateral periapical lesions and produced no output category at all for internal/external resorption, retrograde root fillings, or instrument fragments. While individually rare in our dataset, these findings collectively represent an important clinical decision point in CBCT-based endodontic re-evaluation—particularly resorption, where progressive disease may dictate a switch from non-surgical retreatment to extraction. Several research-grade CNNs have demonstrated good detection performance for external root resorption on CBCT (accuracy 0.81–0.97 in laboratory and clinical datasets) [33], but these capabilities are not currently exposed by the commercial Diagnocat^®^ platform. Until such modules are added or the AI’s training distribution is extended to include the lower-prevalence findings, clinicians should not regard Diagnocat^®^ as a comprehensive endodontic screening tool, but rather as a focused adjunct that augments the human reader for specific high-frequency tasks—most clearly missed canal, crown identification, and major root-canal-filling quality flaws.

### 4.6. Strengths

The strengths of the present study reflect a deliberate design intended to produce a methodologically rigorous evaluation. (i) The reference standard relied on the independent assessment of two endodontists with senior-expert adjudication of disagreements, mitigating single-rater bias and producing a consensus label that, while not free from reader uncertainty, is more robust than commonly used single-rater gold standards [26]. (ii) All confidence intervals were estimated by cluster bootstraps with the scan as the resampling unit, preserving the within-scan correlation of teeth that previous studies have ignored. (iii) Threshold optimization, probability calibration (Brier, intercept, slope), and decision-curve analysis were pre-specified rather than added post hoc, in line with current methodological guidance for diagnostic-AI evaluation [29,30]. (iv) Cohen’s κ was complemented by Gwet’s first-order agreement coefficient AC1 to mitigate the well-documented kappa paradox under skewed prevalence [28]. (v) Reporting follows STARD 2015 [26].

### 4.7. Limitations

Limitations should be considered. The single-center, single-device, single-operator-team origin of the dataset limits generalizability to other CBCT vendors, voxel sizes and reconstruction kernels—a recurring issue across published dental AI evaluations [15]. The retrospective, archive-based design may have selected for cases of higher clinical complexity, which could in turn bias the apical-lesion prevalence (72%) upward relative to the general adult RCT population [2]. Apicoectomy and resorption findings were rare (n = 30 and n = 19, respectively), producing wide confidence intervals that limit firm conclusions for these findings. A single tooth (0.3%) showed a discordance between its Estrela class (s4) and the binary apical-lesion label after consensus adjudication; this was retained as recorded and is the source of the off-by-one count between the corresponding rows of Table 1 and Table 5. Finally, all subgroup analyses were exploratory and unadjusted for multiplicity; the GEE interaction *p*-values should be interpreted as hypothesis-generating rather than confirmatory.

A key limitation of this study is its retrospective single-center design. All CBCT examinations were acquired in one private practice using a single CBCT system and local imaging protocols. Therefore, the findings should be interpreted as reflecting performance under these specific conditions rather than broad clinical applicability. In addition, the referral-based cohort showed a high prevalence of apical lesions, which may have influenced predictive values and decision-curve analyses. Multicenter studies using different CBCT systems, imaging protocols, and patient populations are needed to confirm the generalizability of these results.

An additional limitation concerns the 25 root-canal-treated teeth (6.5%) that were not recognized as teeth by Diagnocat^®^ and therefore did not generate analyzable AI outputs. From a clinical perspective, such recognition failures should be regarded as a relevant component of overall system performance because a screening tool cannot assist the clinician if a tooth is not identified in the first place. Importantly, recognition failure was not restricted to low-quality CBCT examinations. Most unrecognized teeth originated from scans classified as very good or clinically good with respect to image quality, whereas only a small proportion originated from high-noise scans. A large majority of the affected teeth were restored with full-coverage crowns, suggesting that restorative complexity may contribute to this failure mode. Consequently, the diagnostic-accuracy estimates reported for the successfully processed subset may overestimate the real-world utility of the software.

Another limitation relates to the low prevalence of several evaluated findings. While the dataset provided adequate numbers for the principal analyses of apical lesions, root-filling quality parameters, missed canals, and crowns, other findings such as apicoectomy were represented by fewer positive cases, resulting in wider confidence intervals and less precise performance estimates. Furthermore, internal resorption, external resorption, retrograde fillings, and instrument fragments occurred too infrequently, or were not supported by the software output, to permit formal diagnostic-accuracy analyses. Consequently, observations regarding these findings should be considered preliminary and interpreted with caution.

### 4.8. Implications for Clinical Practice and Future Work

In clinical practice, the present results support the use of Diagnocat^®^ as a screening adjunct to—not a replacement for—a trained endodontic reader on CBCT, particularly for missed canal flagging, crown identification and root canal filling quality detection, where the platform now exhibits levels of agreement (κ 0.6–0.8) generally accepted as good and up to excellent. For screening of apical lesions, the platform is most useful when interpreted in conjunction with lesion size: lesions ≥ 1 mm are detected with high sensitivity, but sub-millimeter lesions remain a known blind spot for both the human eye and the AI [12,18]. This work highlights the described need for conducting deployment studies for such AI-based dental applications to translate and implement them into dental practice [34]. From a deployment perspective, our threshold analysis suggests that institutions adopting the platform should consider task-specific decision thresholds rather than the manufacturer-fixed 0.50 cut-off; this would raise sensitivity for missed canal and overfilled root canal at acceptable specificity cost. From a research perspective, three priorities emerge: (i) prospective multi-center, multi-device validation of the same module against the same consensus framework; (ii) extension of the Diagnocat^®^ output to the four endodontic findings currently not modelled; and (iii) head-to-head comparison of Diagnocat^®^ as an unaided AI versus AI-assisted endodontic reading, to quantify the actual gain in the clinical workflow that current AUC-based metrics merely promise.

## 5. Conclusions

In a clinically representative cohort of 358 root canal treated teeth, Diagnocat^®^ AI demonstrated very high diagnostic performance for missed canals (AUC 0.984), crown detection (AUC 0.893) and root canal filling quality (short root filling AUC 0.902, overfilled root canal AUC 0.917) on CBCT, with weaker but still useful performance for apical-lesion detection (AUC 0.864). Detection of apical lesions was strongly dependent on lesion size and Estrela CBCT-PAI severity class, with sub-millimeter lesions largely missed and sensitivity exceeding 80% measuring 1–5 mm, whereas performance was lower in the ≥5 mm stratum. A negative AI output should not be interpreted as ruling out clinically relevant pathology.

Re-tuning the manufacturer-fixed 0.50 probability threshold yielded clinically meaningful sensitivity gains, especially for missed canal (69.6 → 97.5%), and decision curve analysis confirmed positive net benefit for missed canal and root canal filling quality findings across the entire clinically plausible threshold range. No consistent decline in performance across the SNR categories investigated was observed; however, the limited number of high-noise cases warrant cautious interpretation.

Diagnocat^®^ can therefore be recommended as a focused screening adjunct in CBCT-based endodontic interpretation for high-frequency, high contrast findings, with cautious clinical interpretation for early apical disease and an explicit acknowledgment that lateral periapical lesions, internal and external resorption, retrograde root fillings and instrument fragments are not currently part of its analyzable output. Multi-center, multi-device validation, and direct comparison with AI-assisted human reading represent the most informative next steps for translating these diagnostic accuracy findings into a quantified clinical workflow benefit. Although threshold optimization demonstrated the potential to improve diagnostic performance for several findings, the optimized thresholds identified in this study require external validation using a multi-center approach including different devices before clinical implementation could be recommended.

## Figures and Tables

**Table 1:** Cohort and tooth-level characteristics of the analytic sample (n = 358 teeth). Categorical variables are presented as n (%); continuous variables as mean ± SD with median and range. Estrela classification refers to the CBCT periapical index, with the s4–s6 grouping representing periapical osteitis with cortical bone destruction or expansion. Image-quality categories are derived from the signal-to-noise ratio (SNR = mean ROI grey value/SD); the manufacturer-suggested cut-offs are >10 (very good), 6–10 (clinically good), 3–6 (moderate), <3 (high noise).

Characteristic	Total (n = 358 Teeth)
**Patient/scan level**	
Patients (n)	177
CBCT scans (n)	177
Patients in analysis sample	167
Scans in analysis sample	167
Teeth per scan, median (IQR)	1 (1–3)
Teeth per scan, range	1–8
Tooth level	
Total root-canal-treated teeth (full)	383
Teeth recognized by Diagnocat^®^	358 (93.5)
Teeth excluded (not recognized)	25 (6.5)
Jaw—Maxilla	234 (65.4)
Jaw—Mandible	124 (34.6)
Tooth class—Anterior	67 (18.7)
Tooth class—Premolar	123 (34.4)
Tooth class—Molar	168 (46.9)
FDI group—Incisor	46 (12.8)
FDI group—Canine	21 (5.9)
FDI group—Premolar	123 (34.4)
FDI group—Molar	168 (46.9)
Acquisition	
FOV—4 × 4 cm	67 (18.7)
FOV—10 × 4 cm	2 (0.6)
FOV—10 × 8 cm	289 (80.7)
Image quality (SNR category)	
Very good	103 (28.8)
Clinically good	175 (48.9)
Moderate	69 (19.3)
High noise	11 (3.1)
Continuous measures	
Noise—MEAN gray value (ROI)	1285 ± 429 (median 1222, range 404–2632)
Noise—SD gray value (ROI)	161 ± 64 (median 144, range 92–580)
Signal-to-noise ratio (MEAN/SD)	8.85 ± 3.89 (median 8.08, range 1.68–22.15)
Anatomical canals (gold count)	2.3 ± 1.2 (median 2.0, range 1.0–5.0)
Lesion diameter, mm (all teeth)	1.14 ± 1.67 (median 0.60, range 0.00–13.00)
Lesion diameter, mm (lesion+ teeth)	1.58 ± 1.78 (median 1.10, range 0.10–13.00)
Estrela CBCT-PAI	
s0	99 (27.7)
s1	78 (21.8)
s2	49 (13.7)
s3	69 (19.3)
s4	45 (12.6)
s5	14 (3.9)
s6	4 (1.1)
Grouped: s0–s3	295 (82.4)
Grouped: s4–s6	63 (17.6)
Pre-existing restorations	
Tooth crowned (gold)	210 (58.7)
Apicoectomy (gold)	30 (8.4)
Retrograde filling (gold)	16 (4.5)
Instrument fragment (gold)	1 (0.3)
Internal resorption (gold)	5 (1.4)
External resorption (gold)	14 (3.9)

**Table 2:** Diagnostic performance of Diagnocat^®^ AI per tooth (n = 358) using the manufacturer-fixed probability threshold of 0.50. Prevalence is the proportion of gold-standard-positive teeth. TP/FN/FP/TN = true positives, false negatives, false positives, true negatives. Sensitivity, specificity, positive (PPV) and negative (NPV) predictive values, and accuracy are reported with 95% cluster-bootstrap CIs (2000 resamples; cluster = scan). The AUC is computed on the continuous probability score; Cohen κ and Gwet AC1 quantify agreement on the binarized AI label vs. the consensus gold standard. Youden J = sensitivity + specificity − 1.

Finding	n	Prev (%)	TP/FN/FP/TN	Sensitivity (% [95% CI])	Specificity (% [95% CI])	PPV (% [95% CI])	NPV (% [95% CI])	Accuracy (% [95% CI])	AUC (95% CI)	Cohen κ (95% CI)	Gwet AC1 (95% CI)	Youden J (95% CI)
Apical lesion	358	72.1	158/100/5/95	61.2 (54.9–67.5)	95.0 (90.0–99.0)	96.9 (93.5–99.4)	48.7 (41.3–55.9)	70.7 (65.6–75.7)	0.864 (0.827–0.900)	0.436 (0.356–0.519)	0.431 (0.330–0.533)	0.562 (0.485–0.639)
Short root filling	358	43.0	114/40/26/178	74.0 (65.8–81.4)	87.3 (83.0–91.5)	81.4 (75.0–87.5)	81.7 (75.9–87.1)	81.6 (77.3–85.4)	0.902 (0.866–0.932)	0.620 (0.531–0.699)	0.643 (0.558–0.719)	0.613 (0.523–0.695)
Voids in root filling	358	27.7	59/40/56/203	59.6 (48.0–70.8)	78.4 (72.5–84.1)	51.3 (40.7–61.8)	83.5 (77.8–88.6)	73.2 (67.6–78.5)	0.761 (0.691–0.823)	0.362 (0.237–0.481)	0.538 (0.431–0.637)	0.380 (0.252–0.508)
Missed canal	358	22.1	55/24/1/278	69.6 (58.7–79.4)	99.6 (98.8–100.0)	98.2 (93.8–100.0)	92.1 (88.8–94.9)	93.0 (90.2–95.5)	0.984 (0.972–0.993)	0.773 (0.681–0.851)	0.899 (0.856–0.936)	0.693 (0.585–0.790)
Overfilled root canal	358	17.0	42/19/27/270	68.9 (57.8–78.7)	90.9 (87.5–94.2)	60.9 (49.3–72.9)	93.4 (90.2–96.1)	87.2 (83.5–90.5)	0.917 (0.876–0.953)	0.568 (0.461–0.670)	0.817 (0.757–0.869)	0.598 (0.485–0.705)
Apicoectomy	358	8.4	8/22/0/328	26.7 (9.5–45.2)	100.0 (100.0–100.0)	100.0 (100.0–100.0)	93.7 (90.9–96.3)	93.9 (91.1–96.4)	0.857 (0.775–0.928)	0.400 (0.165–0.600)	0.932 (0.899–0.961)	0.267 (0.095–0.452)
Crown	358	58.7	199/11/29/119	94.8 (91.3–97.7)	80.4 (73.5–86.8)	87.3 (82.3–91.5)	91.5 (85.4–96.3)	88.8 (85.3–92.1)	0.893 (0.851–0.932)	0.765 (0.688–0.834)	0.787 (0.717–0.851)	0.752 (0.674–0.824)

**Table 3:** Sensitivity, specificity, predictive values, accuracy, and Youden J at four probability thresholds (0.30; the manufacturer-fixed 0.50; 0.70; and the data-driven Youden-optimal value) per finding. The Youden-optimal threshold is the probability that maximizes sensitivity + specificity − 1 on the empirical ROC.

Finding	Threshold	Sens (%)	Spec (%)	PPV (%)	NPV (%)	Acc (%)	Youden J
Apical lesion	0.30	64.7	95.0	97.1	51.1	73.2	0.597
Apical lesion	0.50 (default)	61.2	95.0	96.9	48.7	70.7	0.562
Apical lesion	0.70	59.7	95.0	96.9	47.7	69.6	0.547
Apical lesion	Youden-opt (0.07)	70.5	93.0	96.3	55.0	76.8	0.635
Short root filling	0.30	84.4	81.9	77.8	87.4	83.0	0.663
Short root filling	0.50 (default)	74.0	87.3	81.4	81.7	81.6	0.613
Short root filling	0.70	64.9	92.6	87.0	77.8	80.7	0.576
Short root filling	Youden-opt (0.34)	83.1	85.8	81.5	87.1	84.6	0.689
Voids in root filling	0.30	69.7	66.8	44.5	85.2	67.6	0.365
Voids in root filling	0.50 (default)	59.6	78.4	51.3	83.5	73.2	0.38
Voids in root filling	0.70	47.5	84.9	54.7	80.9	74.6	0.324
Voids in root filling	Youden-opt (0.19)	79.8	60.2	43.4	88.6	65.6	0.4
Missed canal	0.30	83.5	98.9	95.7	95.5	95.5	0.825
Missed canal	0.50 (default)	69.6	99.6	98.2	92.1	93.0	0.693
Missed canal	0.70	60.8	100.0	100.0	90.0	91.3	0.608
Missed canal	Youden-opt (0.09)	97.5	88.5	70.6	99.2	90.5	0.86
Overfilled root canal	0.30	83.6	88.6	60.0	96.3	87.7	0.722
Overfilled root canal	0.50 (default)	68.9	90.9	60.9	93.4	87.2	0.598
Overfilled root canal	0.70	63.9	96.0	76.5	92.8	90.5	0.599
Overfilled root canal	Youden-opt (0.15)	91.8	87.2	59.6	98.1	88.0	0.79
Apicoectomy	0.30	53.3	95.4	51.6	95.7	91.9	0.488
Apicoectomy	0.50 (default)	26.7	100.0	100.0	93.7	93.9	0.267
Apicoectomy	0.70	10.0	100.0	100.0	92.4	92.5	0.1
Apicoectomy	Youden-opt (0.16)	83.3	71.6	21.2	97.9	72.6	0.55
Crown	0.30	96.2	80.4	87.4	93.7	89.7	0.766
Crown	0.50 (default)	94.8	80.4	87.3	91.5	88.8	0.752
Crown	0.70	94.8	80.4	87.3	91.5	88.8	0.752
Crown	Youden-opt (0.30)	96.2	80.4	87.4	93.7	89.7	0.766

**Table 4:** Subgroup performance (sensitivity and specificity, with 95% cluster-bootstrap CIs) for each of the seven analyzable findings stratified by jaw, tooth class, FOV, and image-quality category. The right-hand column reports the *p*-value for the AI-probability × subgroup interaction term in a GEE logistic regression with exchangeable working correlation and robust standard errors. n (n+) = total stratum size with the number of gold-positive teeth. NE = non-estimable because of sparse events and complete or quasi-complete separation. Interaction tests affected by separation were not interpreted.

Finding	Subgroup	Level	n (n+)	Sens % (95% CI)	Spec % (95% CI)	*p*-Int (GEE)
Apical lesion	Jaw	Maxilla	234 (165)	58.8 (50.8–66.4)	92.8 (85.5–98.6)	0.038
Apical lesion	Jaw	Mandible	124 (93)	65.6 (54.5–75.5)	100.0 (100.0–100.0)	0.038
Apical lesion	Tooth class	Anterior	67 (44)	52.3 (37.8–70.0)	95.7 (85.7–100.0)	0.781
Apical lesion	Tooth class	Premolar	123 (82)	48.8 (37.7–60.4)	95.1 (87.1–100.0)	0.781
Apical lesion	Tooth class	Molar	168 (132)	72.0 (64.2–79.1)	94.4 (83.3–100.0)	0.781
Apical lesion	FOV	4 × 4	67 (47)	72.3 (61.0–83.3)	100.0 (100.0–100.0)	0.367
Apical lesion	FOV	10 × 8	289 (211)	58.8 (51.9–65.6)	93.6 (87.7–98.7)	0.367
Apical lesion	Image quality	Very good	103 (72)	66.7 (55.2–76.6)	96.8 (89.7–100.0)	0.997
Apical lesion	Image quality	Clinically good	175 (127)	58.3 (50.0–67.2)	95.8 (88.1–100.0)	0.997
Apical lesion	Image quality	Moderate	69 (53)	56.6 (41.9–70.2)	93.8 (78.6–100.0)	0.997
Apical lesion	Image quality	High noise	11 (6)	100.0 (100.0–100.0)	80.0 (66.7–100.0)	0.997
Short root filling	Jaw	Maxilla	234 (96)	74.0 (63.9–83.2)	86.2 (80.5–91.1)	0.368
Short root filling	Jaw	Mandible	124 (58)	74.1 (61.8–85.0)	89.4 (82.1–95.6)	0.368
Short root filling	Tooth class	Anterior	67 (24)	50.0 (29.4–68.2)	95.3 (87.8–100.0)	0.553
Short root filling	Tooth class	Premolar	123 (41)	68.3 (54.5–81.1)	91.5 (85.1–97.1)	0.553
Short root filling	Tooth class	Molar	168 (89)	83.1 (73.9–91.1)	78.5 (68.7–86.5)	0.553
Short root filling	FOV	4 × 4	67 (31)	74.2 (57.7–89.3)	75.0 (61.8–87.9)	0.064
Short root filling	FOV	10 × 8	289 (122)	74.6 (64.4–83.0)	89.8 (85.2–93.9)	0.064
Short root filling	Image quality	Very good	103 (47)	68.1 (52.5–81.3)	80.4 (70.0–90.7)	<0.001
Short root filling	Image quality	Clinically good	175 (71)	76.1 (63.2–86.3)	91.3 (86.0–96.3)	<0.001
Short root filling	Image quality	Moderate	69 (31)	74.2 (54.2–89.7)	86.8 (75.8–96.4)	<0.001
Short root filling	Image quality	High noise	11 (5)	100.0 (100.0–100.0)	83.3 (33.3–100.0)	<0.001
Voids in root filling	Jaw	Maxilla	234 (53)	52.8 (37.7–68.8)	77.9 (71.2–84.6)	0.047
Voids in root filling	Jaw	Mandible	124 (46)	67.4 (53.1–81.6)	79.5 (69.4–89.1)	0.047
Voids in root filling	Tooth class	Anterior	67 (18)	44.4 (15.4–71.4)	93.9 (86.4–100.0)	0.287
Voids in root filling	Tooth class	Premolar	123 (27)	51.9 (30.8–72.4)	83.3 (74.4–91.1)	0.287
Voids in root filling	Tooth class	Molar	168 (54)	68.5 (55.2–80.4)	67.5 (57.1–77.2)	0.287
Voids in root filling	FOV	4 × 4	67 (20)	60.0 (40.9–81.0)	72.3 (58.1–83.9)	0.972
Voids in root filling	FOV	10 × 8	289 (78)	60.3 (45.8–72.7)	79.6 (73.2–86.3)	0.972
Voids in root filling	Image quality	Very good	103 (24)	62.5 (42.3–81.0)	75.9 (64.9–85.9)	0.618
Voids in root filling	Image quality	Clinically good	175 (52)	51.9 (33.3–68.3)	82.9 (73.8–90.4)	0.618
Voids in root filling	Image quality	Moderate	69 (22)	72.7 (47.8–90.5)	74.5 (57.9–88.1)	0.618
Voids in root filling	Image quality	High noise	11 (1)	100.0 (100.0–100.0)	60.0 (28.6–90.0)	0.618
Missed canal	Jaw	Maxilla	234 (57)	68.4 (55.5–80.7)	99.4 (98.2–100.0)	0.372
Missed canal	Jaw	Mandible	124 (22)	72.7 (54.2–90.9)	100.0 (100.0–100.0)	0.372
Missed canal	Tooth class	Anterior	67 (0)	—	100.0 (100.0–100.0)	—
Missed canal	Tooth class	Premolar	123 (6)	50.0 (0.0–100.0)	100.0 (100.0–100.0)	—
Missed canal	Tooth class	Molar	168 (73)	71.2 (60.0–82.9)	98.9 (96.6–100.0)	—
Missed canal	FOV	4 × 4	67 (20)	75.0 (52.9–93.8)	100.0 (100.0–100.0)	0.844
Missed canal	FOV	10 × 8	289 (59)	67.8 (55.9–80.4)	99.6 (98.6–100.0)	0.844
Missed canal	Image quality	Very good	103 (16)	62.5 (36.8–88.9)	100.0 (100.0–100.0)	<0.001
Missed canal	Image quality	Clinically good	175 (44)	72.7 (59.2–85.1)	100.0 (100.0–100.0)	<0.001
Missed canal	Image quality	Moderate	69 (17)	70.6 (46.7–92.3)	98.1 (94.0–100.0)	<0.001
Missed canal	Image quality	High noise	11 (2)	50.0 (0.0–100.0)	100.0 (100.0–100.0)	<0.001
Overfilled root canal	Jaw	Maxilla	234 (42)	71.4 (56.9–84.8)	91.1 (87.0–95.0)	0.636
Overfilled root canal	Jaw	Mandible	124 (19)	63.2 (38.9–84.6)	90.5 (84.6–95.6)	0.636
Overfilled root canal	Tooth class	Anterior	67 (10)	80.0 (50.0–100.0)	94.7 (88.1–100.0)	0.643
Overfilled root canal	Tooth class	Premolar	123 (24)	58.3 (38.1–77.8)	94.9 (90.1–98.9)	0.643
Overfilled root canal	Tooth class	Molar	168 (27)	74.1 (56.5–89.3)	86.5 (80.3–92.1)	0.643
Overfilled root canal	FOV	4 × 4	67 (19)	57.9 (35.3–76.5)	81.2 (69.8–92.0)	0.002
Overfilled root canal	FOV	10 × 8	289 (42)	73.8 (61.5–87.1)	92.7 (89.3–95.9)	0.002
Overfilled root canal	Image quality	Very good	103 (23)	73.9 (54.2–90.0)	90.0 (82.8–95.9)	0.021
Overfilled root canal	Image quality	Clinically good	175 (29)	69.0 (54.5–85.0)	89.0 (83.8–94.2)	0.021
Overfilled root canal	Image quality	Moderate	69 (9)	55.6 (25.0–83.3)	96.7 (92.1–100.0)	0.021
Overfilled root canal	Image quality	High noise	11 (0)	—	90.9 (75.0–100.0)	0.021
Apicoectomy	Jaw	Maxilla	234 (20)	25.0 (7.1–47.1)	100.0 (100.0–100.0)	0.570
Apicoectomy	Jaw	Mandible	124 (10)	30.0 (0.0–60.0)	100.0 (100.0–100.0)	0.570
Apicoectomy	Tooth class	Anterior	67 (12)	16.7 (0.0–44.4)	100.0 (100.0–100.0)	0.351
Apicoectomy	Tooth class	Premolar	123 (9)	55.6 (14.3–87.5)	100.0 (100.0–100.0)	0.351
Apicoectomy	Tooth class	Molar	168 (9)	11.1 (0.0–36.4)	100.0 (100.0–100.0)	0.351
Apicoectomy	FOV	4 × 4	67 (4)	0.0 (0.0–0.0)	100.0 (100.0–100.0)	0.890
Apicoectomy	FOV	10 × 8	289 (25)	32.0 (11.1–53.9)	100.0 (100.0–100.0)	0.890
Apicoectomy	Image quality	Very good	103 (8)	25.0 (0.0–60.0)	100.0 (100.0–100.0)	NE
Apicoectomy	Image quality	Clinically good	175 (18)	33.3 (10.0–61.9)	100.0 (100.0–100.0)	NE
Apicoectomy	Image quality	Moderate	69 (4)	0.0 (0.0–0.0)	100.0 (100.0–100.0)	NE
Apicoectomy	Image quality	High noise	11 (0)	—	100.0 (100.0–100.0)	NE
Crown	Jaw	Maxilla	234 (142)	93.0 (88.4–97.2)	81.5 (72.0–90.1)	0.308
Crown	Jaw	Mandible	124 (68)	98.5 (95.1–100.0)	78.6 (67.9–89.5)	0.308
Crown	Tooth class	Anterior	67 (50)	94.0 (86.5–100.0)	94.1 (78.6–100.0)	0.372
Crown	Tooth class	Premolar	123 (72)	93.1 (87.3–98.5)	80.4 (68.7–90.4)	0.372
Crown	Tooth class	Molar	168 (88)	96.6 (92.6–100.0)	77.5 (68.3–86.4)	0.372
Crown	FOV	4 × 4	67 (24)	91.7 (80.0–100.0)	76.7 (62.8–89.2)	0.491
Crown	FOV	10 × 8	289 (184)	95.1 (91.5–98.1)	81.9 (74.1–88.4)	0.491
Crown	Image quality	Very good	103 (57)	96.5 (90.9–100.0)	76.1 (62.2–88.2)	0.859
Crown	Image quality	Clinically good	175 (94)	93.6 (87.9–98.0)	80.2 (70.1–89.0)	0.859
Crown	Image quality	Moderate	69 (48)	93.8 (86.0–100.0)	90.5 (79.2–100.0)	0.859
Crown	Image quality	High noise	11 (11)	100.0 (100.0–100.0)	—	0.859

**Table 5:** Diagnocat^®^ sensitivity for apical lesions stratified by Estrela CBCT periapical index. Estrela s0 is by definition lesion-negative and is shown for completeness only. Confidence intervals are 95% cluster-bootstrap intervals over scan-level resamples. n teeth = total number of teeth in the stratum; Lesion+ (n) = number of gold-standard-positive teeth contributing to the sensitivity estimate.

Stratum	n Teeth	Lesion+ (n)	AI Sensitivity (% [95% CI])
Estrela s0	99	0	—
Estrela s1	78	78	24.4 (14.3–34.1)
Estrela s2	49	49	61.2 (47.0–74.0)
Estrela s3	69	69	82.6 (73.9–91.7)
Estrela s4	45	44	86.4 (76.1–95.5)
Estrela s5	14	14	78.6 (57.1–100.0)
Estrela s6	4	4	75.0 (50.0–100.0)
Grouped s0–s3	295	196	54.1 (46.8–61.4)
Grouped s4–s6	63	62	83.9 (75.0–92.4)

**Table 6:** Diagnocat^®^ sensitivity for apical lesions stratified by lesion diameter (gold-positive teeth only). Confidence intervals are 95% cluster-bootstrap intervals over scan-level resamples.

Stratum	Lesion+ (n)	AI Sensitivity (% [95% CI])
<1 mm	110	33.6 (24.5–42.5)
1–<3 mm	113	81.4 (73.4–88.6)
3–<5 mm	24	91.7 (80.7–100.0)
≥5 mm	11	63.6 (36.4–90.0)

## Data Availability

The raw anonymized data generated and analyzed in this study are not publicly archived but are available from the corresponding author upon reasonable request for research purposes. Because the dataset contains anonymized clinical imaging information, access can be granted to qualified researchers in accordance with institutional and ethical guidelines.

## Supplementary Materials

The following supporting information can be downloaded at: https://www.mdpi.com/article/10.3390/jcm15124839/s1, Figure S1: Probability-score histograms per finding, stratified by gold-standard label; Figure S2: Anatomical canal counts—Diagnocat^®^ AI versus consensus gold standard; Figure S3: Apical-lesion detection by Estrela CBCT-PAI class (true-positive/false-negative stacked bars); Table S1: Calibration metrics (Brier score, intercept, slope) per finding; Table S2: Sensitivity analyses—worst-case full cohort (n = 383) and per-patient subset (n = 167).
