# Supplementary material for: Diagnostic Performance of AI-Based Cloud Software Regarding the Detection of Endodontic Findings on CBCT: A Single-Centre Cross-Sectional Validation Study
Source: J Clin Med. 2026 Jun 22;15(12):4839. doi: 10.3390/jcm15124839 (PMC13302509; doi:10.3390/jcm15124839)

## Supplementary Figure S1

**Figure S1.** Probability-score histograms per finding, stratified by gold-standard label (gold-negative in light blue, gold-positive in orange). The vertical dashed line marks the manufacturer-fixed 0.50 decision threshold. Bimodal distributions with strong separation (e.g. missed canal, crown) correspond to high-AUC findings; substantial overlap between gold-positive and gold-negative distributions characterises the lower-AUC findings.

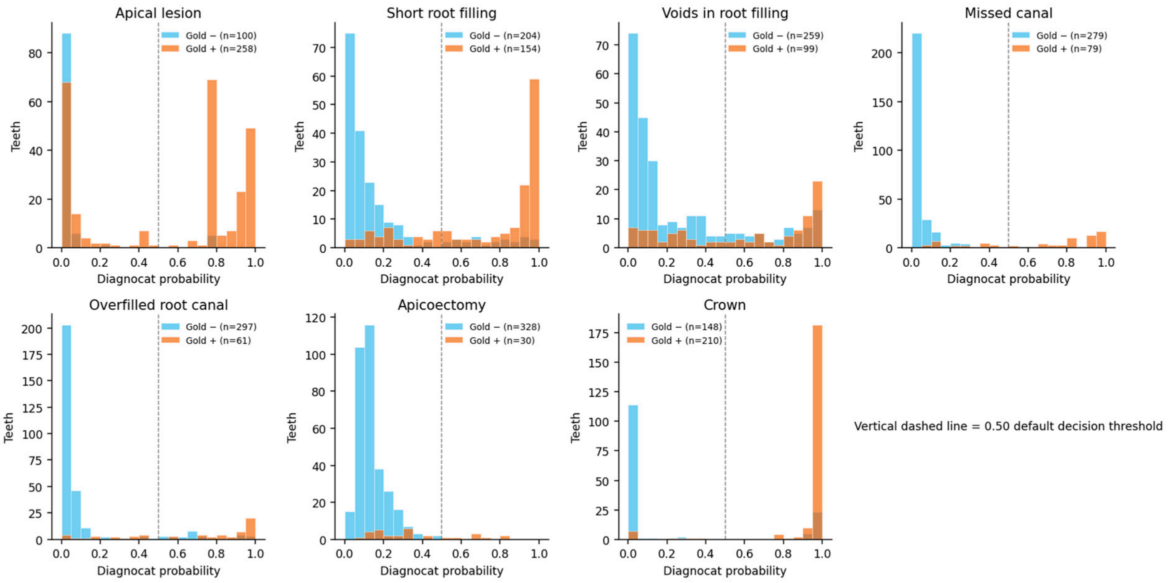

Supplement: Supplementary file 1 [file jcm-15-04839-s001.zip › Supplementary_Figure_S1.pdf]
