# Supplementary material for: Diagnostic Performance of AI-Based Cloud Software Regarding the Detection of Endodontic Findings on CBCT: A Single-Centre Cross-Sectional Validation Study
Source: J Clin Med. 2026 Jun 22;15(12):4839. doi: 10.3390/jcm15124839 (PMC13302509; doi:10.3390/jcm15124839)

Supplementary Figure S2

Figure S2. Anatomical canal counts: Diagnocat AI versus consensus gold (n = 358 teeth, jittered for visibility). The dashed line is the line of identity. Annotation indicates exact-agreement rate, the proportion within ±1 canal, the Spearman rank correlation, and the linearly weighted Cohen κ.

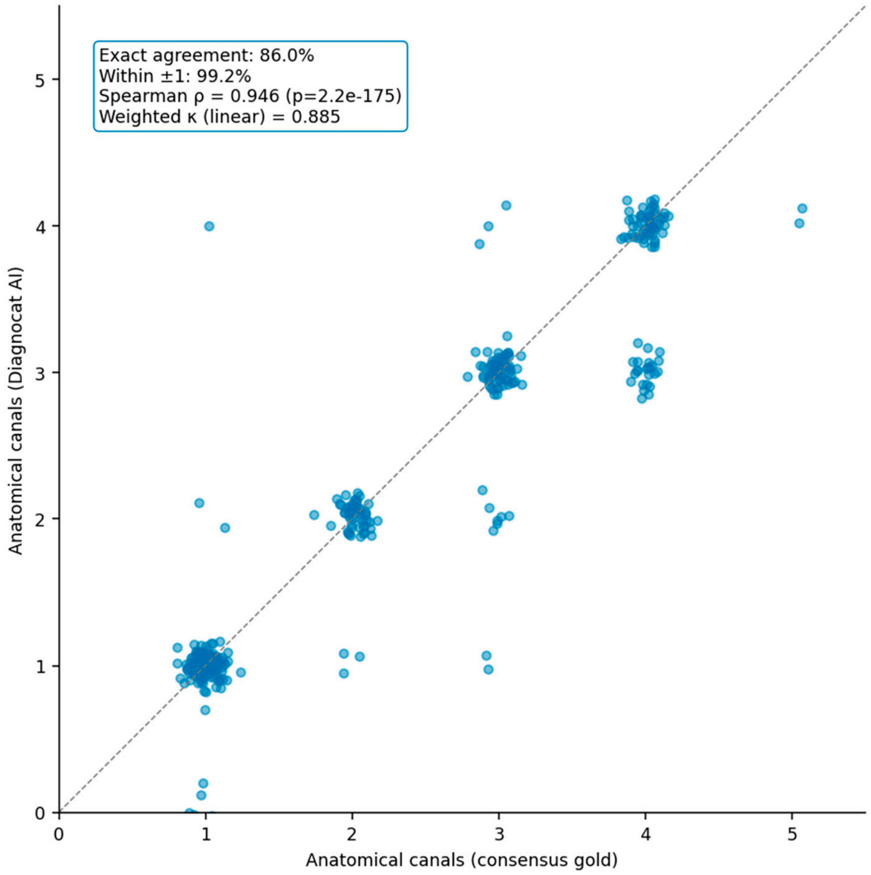

Supplement: Supplementary file 1 [file jcm-15-04839-s001.zip › Supplementary_Figure_S2.pdf]
