# Supplementary material for: Diagnostic Performance of AI-Based Cloud Software Regarding the Detection of Endodontic Findings on CBCT: A Single-Centre Cross-Sectional Validation Study
Source: J Clin Med. 2026 Jun 22;15(12):4839. doi: 10.3390/jcm15124839 (PMC13302509; doi:10.3390/jcm15124839)

Supplementary Figure S3

Figure S3. Stacked bar chart of apical-lesion detection by Estrela class. Each bar represents all gold-positive teeth in the Estrela stratum; the green segment shows true positives (detected) and the orange segment shows false negatives (missed). Sensitivity rises monotonically from Estrela s1 (24%) to s4 (86%), consistent with size and contrast effects discussed in Section 3.5. Estrela s0 is by definition lesion-negative and contributes no bar.

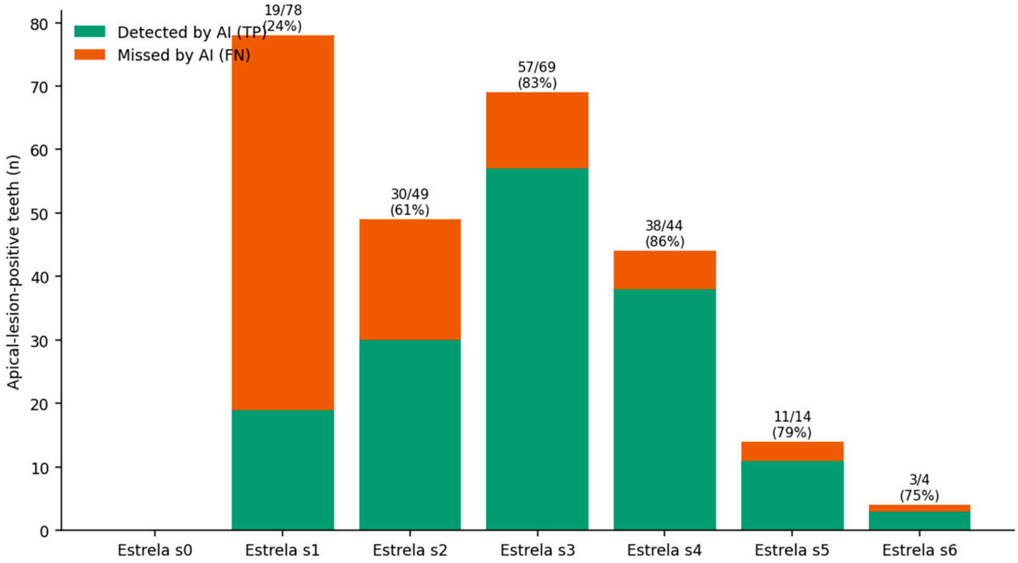

Supplement: Supplementary file 1 [file jcm-15-04839-s001.zip › Supplementary_Figure_S3.pdf]
