# Supplementary material for: Diagnostic Performance of AI-Based Cloud Software Regarding the Detection of Endodontic Findings on CBCT: A Single-Centre Cross-Sectional Validation Study
Source: J Clin Med. 2026 Jun 22;15(12):4839. doi: 10.3390/jcm15124839 (PMC13302509; doi:10.3390/jcm15124839)
Supplement: Supplementary file 1 [file jcm-15-04839-s001.zip › Supplementary_Table_S1.pdf]

Supplementary Table S1

Table S1. Calibration metrics per finding. The Brier score is the mean squared difference between predicted probability and observed outcome (lower is better; 0 = perfect, 0.25 = uninformative for prevalences near 0.5). Calibration intercept and slope are obtained by logistic recalibration of the gold-standard label on logit(Diagnocat probability); a perfectly calibrated model has intercept = 0 and slope = 1.

Finding	n	n positive	Brier score	Calibration intercept (logit)	Calibration slope
Apical lesion	358	258	0.266	1.78	0.295
Short root filling	358	154	0.127	0.018	0.746
Voids in root filling	358	99	0.201	-0.782	0.4
Missed canal	358	79	0.049	2.013	1.773
Overfilled root canal	358	61	0.083	-0.767	0.651
Apicoectomy	358	30	0.06	0.655	2.063
Crown	358	210	0.103	0.514	0.311
