# Supplementary material for: Diagnostic Performance of AI-Based Cloud Software Regarding the Detection of Endodontic Findings on CBCT: A Single-Centre Cross-Sectional Validation Study
Source: J Clin Med. 2026 Jun 22;15(12):4839. doi: 10.3390/jcm15124839 (PMC13302509; doi:10.3390/jcm15124839)
Supplement: Supplementary file 1 [file jcm-15-04839-s001.zip › Supplementary_Table_S2.pdf]

Supplementary Table S2

Table S2. Pre-specified sensitivity analyses. (a) Worst-case full-cohort analysis (n = 383 teeth): the 25 teeth not recognised by Diagnocat are treated as AI-negative for every finding. (b) Per-patient analysis: one randomly selected tooth per scan (n = 167 teeth). For both scenarios the same metrics as in the primary Table 2 are reported using the manufacturer-fixed 0.50 threshold.

Finding	Scenario	n / n+	Sens %	Spec %	PPV %	NPV %	Acc %	AUC	κ
Apical lesion	Worst-case (n=383)	383 / 278	56.8	95.2	96.9	45.5	67.4	0.818	0.388
Short root filling	Worst-case (n=383)	383 / 166	68.7	88.0	81.4	78.6	79.6	0.845	0.578
Voids in root filling	Worst-case (n=383)	383 / 105	56.2	79.9	51.3	82.8	73.4	0.735	0.35
Missed canal	Worst-case (n=383)	383 / 85	64.7	99.7	98.2	90.8	91.9	0.919	0.733
Overfilled root canal	Worst-case (n=383)	383 / 65	64.6	91.5	60.9	92.7	86.9	0.868	0.548
Apicoectomy	Worst-case (n=383)	383 / 33	24.2	100.0	100.0	93.3	93.5	0.79	0.369
Crown	Worst-case (n=383)	383 / 229	86.9	81.2	87.3	80.6	84.6	0.848	0.68
Apical lesion	One tooth per scan	167 / 122	61.5	97.8	98.7	48.4	71.3	0.862	0.448
Short root filling	One tooth per scan	167 / 76	65.8	82.4	75.8	74.3	74.9	0.868	0.487
Voids in root filling	One tooth per scan	167 / 45	57.8	75.4	46.4	82.9	70.7	0.761	0.308
Missed canal	One tooth per scan	167 / 38	76.3	100.0	100.0	93.5	94.6	0.983	0.833
Overfilled root canal	One tooth per scan	167 / 32	68.8	89.6	61.1	92.4	85.6	0.918	0.557
Apicoectomy	One tooth	167 / 14	14.3	100.0	100.0	92.7	92.8	0.768	0.234

	per scan								
Crown	One tooth per scan	167 / 89	93.3	78.2	83.0	91.0	86.2	0.874	0.721
